# Artificial Intelligence and Non-Destructive Testing Data to Assess Concrete Sustainability of Civil Engineering Infrastructures

**DOI:** 10.3390/ma18040826

**Published:** 2025-02-13

**Authors:** Cédric Baudrit, Sylvain Dufau, Géraldine Villain, Zoubir Mehdi Sbartaï

**Affiliations:** 1 I2M, CNRS, UMR 5295, INRAE, Université de Bordeaux, F-33400 Talence, France; cedric.baudrit@u-bordeaux.fr (C.B.); sylvain.dufau@u-bordeaux.fr (S.D.); 2 Univ Gustave Eiffel, MAST-LAMES, Campus de Nantes, F-44344 Bouguenais, France; geraldine.villain@univ-eiffel.fr

**Keywords:** Artificial Neural Network (ANN), Random Forest (RF), Machine Learning, SHapley Additive exPlanation (SHAP)

## Abstract

The sustainable development and preservation of natural resources have highlighted the critical need for the effective maintenance of civil engineering infrastructures. Recent advancements in technology and data digitization enable the acquisition of data from sensors on structures like bridges, tunnels, and energy production facilities. This paper explores “smart” uses of these data to optimize maintenance actions through interdisciplinary approaches, integrating artificial intelligence in civil engineering. Corrosion, a key factor affecting infrastructure health, underscores the need for robust predictive maintenance models. Supervised Machine Learning regression methods, particularly Random Forest (RF) and Artificial Neural Networks (ANNs), are investigated for predicting structural properties based on Non-Destructive Testing (NDT) data. The dataset includes various measurements such as ultrasonic, electromagnetic, and electrical on concrete samples. This study compares the performances of RF and ANN in predicting concrete characteristics, like compressive strength, elastic modulus, porosity, density, and saturation rate. The results show that, while both models exhibit strong predictive capabilities, RF generally outperforms ANN in most metrics. Additionally, SHapley Additive exPlanation (SHAP) provides insights into model decisions, ensuring transparency and interpretability. This research emphasizes the potential of integrating Machine Learning with empirical and mechanical methods to enhance infrastructure maintenance, providing a comprehensive framework for future applications.

## 1. Introduction

The collapse of the Morandi bridge in Genoa in 2018 raised many questions about safety standards. The corrosion of reinforced steel, largely due to insufficient maintenance, contributed significantly to this disaster. In 2019, it was reported that at least 25,000 bridges in France were in poor structural condition [1]. Thanks to technological advances and data digitalization, we can now collect vast amounts of valuable information from sensors monitoring on urban infrastructure (bridges, viaducts, tunnels, etc.) or energy production (wind turbines, nuclear power plants, etc.). In the era of digital transition, the “smart” use of data to optimize and guide maintenance strategies has become crucial.

The health of the structure might depend on a handful of factors and mechanisms to identify and prevent. The most notable of which is corrosion, which is responsible for over 80% of the observed pathologies [2]. It describes a process in which a local initiation of the corrosion starts to consume the reinforcements and propagate, leading to the deterioration of the strength in structures. The physico-chemical nature of the reaction and its locality make its measurement all the more difficult, however it is still possible to detect some related indicators. The water saturation degree of concrete, for instance, plays a significant role in reinforced concrete degradation since it allows aggressive chemical agents such as chloride or carbon dioxide to penetrate the concrete and can result in rebar corrosion. The porosity is also important because if the material is porous, the mechanical resistance and defense against the environment are weaker.

To assess those quantities, the first step is to test the concrete with various methods to gather information. These are classified into two categories:

- Destructive Testing (DT) generally consists of taking a sample from the infrastructure and testing it. The advantage is that it directly provides an outcome that matches the desired investigated properties. Tests for strength, like compressive strength testing, flexural strength testing, or twisting methods [3], are ‘direct’ measurements of the strength. DT is a reliable tool to investigate the infrastructure but contributes to the damage it seeks to measure. Moreover, once the measurement is performed, the sample cannot be reused to measure the evolution of the infrastructure. To assert a diagnosis without damaging the concrete, experts in the field must adopt a more subtle approach by examining the effects that lead to damage;
- Non-Destructive Testing (NDT) refers to the set of techniques available to track the health of the structure. Unlike DT, once the sensors are in place it allows multiple measurements in the same area and through time. Damage is limited as well, which is interesting for a continuous evaluation of the structure. However, NDT needs models of conversion to interpret the measurements into the properties of the structure. The standard EN 13791:2019, for instance, describes the procedure to evaluate strength with indirect methods by combining acoustic velocity with rebound method [4].

While both DT and NDT can enrich the knowledge of the structure, NDT is more suitable for continuous evaluation. That is why effort is put into creating conversion models in the literature. For example, natural laws exist between the velocity of shear and pressure acoustic waves (observables obtainable from piezoelectric sensors) and Young’s modulus E [2]. Mixing law can also be used in certain cases, such as the CRIM model [5,6]. By using the SonReb method, which is the combination of two NDT techniques (ultrasonic pulse velocity “UPV” and Rebound hammer), Alavi et al. [7] used a nonlinear equation to improve the accuracy. They achieved $R^{2}$ of 0.97. In [8], B. Gunes et al. expend the scope of observables used to measure the resistance with methods described in the EN 13791:2019 like the drilling resistance, rebound hammer (type Q), and UPV.

Conversion models are non-univocal, and there is no unique solution to the problem, only models that perform better. Models of conversion are often specific to a material, incomplete and non-univocal. Numerical, empirical, and other models each provide only a partial view of the state of the concrete. All methods, such as ultrasound and rebound hammer [9,10], can be difficult to calibrate. Significant differences in compressive strength prediction models exist in the literature due to these irregularities. For this reason, predicted models cannot be generalized for the different concrete mixes due to the multiple effects of concrete characteristics [11].

To improve the calibration process on real structures for a more understandable prediction, a holistic approach is necessary. To this end, Supervised Machine Learning regression uses data-driven techniques and algorithms to predict or infer new data based on historical data. The more commonly used methods [12,13] are based on learning using Artificial Neural Network on a database gathering NDT measurements to evaluate the moisture content and chloride content as well as the compressive strength [14]. Asteris et al. [15] observe a significant improvement in the prediction when ANN is used. The validity of the results can be sought with complementary analysis of the quality of information by using a method based on fuzzy sets to combine data and evaluate the quality of the results [16]. However, all observables are not necessary to obtain a good performance for ML. This is because, some NDT observables can be less sensitive to the indicator variation and contribute weakly to the prediction. In addition, some observables can yield redundant information not necessary to improve prediction performance [17].

The challenge for optimizing and predicting performance of concrete civil infrastructure is to evaluate concrete properties with NDT techniques. However, many effects reduce the reliability of non-destructive measurement as concrete variability and concrete moisture in the porosity that affect NDT measurement, like ultrasonic waves. Moreover, there is a need for interpretability of these models. Explainability in Machine Learning refers to the capability to comprehend and interpret a model’s decision. It enables stakeholders, such as data scientists, domain experts, and end-users, to understand the reasoning behind a model’s predictions or choices [18,19].

In this paper, a new combination of NDT methods is proposed for improving the diagnosis of concrete structures and facilitating informed decision-making. The combined NDT techniques (such as UPV, GPR, etc.) are selected from the most complementary ones that are sensitive to concrete performance and durability indicators (compressive strength, modulus of elasticity, porosity, and degree of water saturation). This new proposed method of combination uses AI algorithms as ANN and RF for predicting concrete performance and durability indicators. Both methods were tested and validated on a real experimental database. Moreover, the use of SHAP method enables the improvement in model explainability calculated by Machine Learning with different approaches. These encompass feature importance analysis, which pinpoints the most impactful features in the model’s decision-making process, and model-agnostic approaches.

## 2. Materials and Methods

### 2.1. Experimental Dataset

An experimental database [20] of 220 samples that combines 3 complementary physical non-destructive measurements (mechanical, electromagnetic, and electrical) is used [21]. It contains observables measured by NDT methods on concrete samples (as ultrasonic pulse velocity, ultrasonic surface wave velocity, resistivity, etc.) and indicators which are characteristics of concrete (compressive strength, modulus of elasticity, porosity, density, water saturation, and volumetric water content). The concept of mechanical NDT variables (ultrasounds) involves transmitting ultrasonic waves using a transmitter sensor, both on the surface and inside the concrete, to analyze the response over time (or frequency). The velocity of wave propagation will be calculated using the distance between the two sensors and the time taken to reach the second sensor [9].

For the electrical variables, a four-electrode technique was used [22]. For the electromagnetic variables, GPR (ground-penetrating radar) was used, equipped with 1.5 GHz antennas [23,24]. For ultrasound waves, velocity was calculated in direct configuration [25,26]. Table 1 summarizes the observables measured by NDT methods. For the characteristics of concrete, there are five different indicators determined by DT:

- Compressive strength of concrete denoted by Rc (MPa), which is the ultimate failure force applied on the concrete divided by the surface;
- Degree of saturation denoted by Sr (%), which is the volume of water in the concrete divided by the volume of concrete pores;
- Porosity denoted by Poro (%). which is volume of void in the concrete divided by the volume of the concrete;
- Elastic modulus denoted by Estat (MPa), which is the derivative of the stress applied on the concrete with respect to the deformation for three cycles at the third of the rupture stress;
- Density of concrete (kg/m).

**Table 1:** Definition of the observables by type.

Types	Observables	Definition	Unit
Mechanical	MEC1	Surface wave velocity in concrete depth (at frequency 1)	m/s
	MEC2	The surface wave velocity at a concrete (at frequency 2)	m/s
	MEC3	The velocity when the two sensors are placed on two different sides of the concrete	m/s
	MEC4	The vibration frequency using the Impact Echo method	Hz
Electrical	ELE1	Resistivity when electrodes are 5 cm apart	Ohm.m
	ELE2	Resistivity when electrodes are 10 cm apart	Ohm.m
	ELE3	ELE1/ELE2	Ø
Electromagnetical	EM1	Peak-to-peak amplitude of the received GPR signal	Ø
	EM2	The velocity of GPR waves	m/s
	EM3	Time taken by a GPR wave to reach the receiving antenna at fixed distance 1	ns
	EM4	Time taken by a GPR wave to reach the receiving antenna at fixed distance 2	ns

### 2.2. Conversion Models

There is no Supervised Machine Learning method that is universally better than the others. Each method has its own advantages and disadvantages depending on the context, the data, and the objective of the task [27,28]. Linear models (ex: Ridge logistic regression, Stochastic Gradient Descent (SGD)) assume a specific relationship between variables which may yield poor predictions and misleading interpretations if the assumptions do not hold. Kernel machines (ex: Support Vector Machine (SVM) [29]) aim to find an optimal hyperplane that best separates the data points in the input space but require careful choice of kernels to avoid overfitting. Local methods (K-Nearest Neighbors (KNN) and Decision Trees) propose interpretable models with low bias but high variance, making them sensitive to data changes and potentially unstable with small datasets. Ensemble methods (Gradient Boosting and Random Forests) combine a large number of simpler models to achieve high predictive performance by making very few assumptions and by balancing low bias and variance, often using averaging. Other categories or specialized algorithms (e.g., neural networks, probabilistic models like Naive Bayes, etc. [30]) can also be considered extensions or hybrids of these types depending on the context. Faced with small sample data, Random Forest is usually the more robust choice because it is less prone to overfitting compared to many other models, thanks to its ensemble approach and bagging technique, which helps improve model stability and accuracy even with limited data. As RF, ANNs have the ability to learn complex nonlinear relationships from the data through their hidden layers. Both Random Forests (RFs) and Artificial Neural Networks (ANNs) are chosen as multi-variable regression models for their flexibility, high predictive performance, and ease of use in a variety of tasks [31]. However, their high flexibility makes them poorly interpretable, requiring additional tools for analysis (see Section 3.2).

All these machine learning algorithms rely on a model architecture defined by hyperparameters, which influence their behavior and performance, and can be determined using optimization algorithms. Based on the work of Malhotra, R. and Cherukuri, M. (2024) [32], among the most commonly adopted methods, Grid Search (GS) explores a predefined set of hyperparameter values and evaluates the model’s performance for each combination. However, the number of evaluations increases exponentially with the number of hyperparameters, potentially leading to high computational complexity. Random Search offers a simpler alternative to GS by randomly sampling hyperparameter values from predefined ranges, allowing optimal or near-optimal configurations to be discovered more efficiently. Population-based methods, such as genetic algorithms and evolutionary algorithms, use optimization strategies that mimic natural selection to iteratively search for optimal hyperparameter configurations. These methods maintain a population of candidate solutions and use genetic operators, such as crossover and mutation, to generate new configurations with potentially improved performance. However, while machine learning methods rely on hyperparameters, population-based methods introduce additional hyperparameters that need configuration, such as the type of fitness function, population size, and crossover rate. Readers can refer to [33] for an exhaustive list of existing hyperparameter optimization methods and their associated computational complexities. Random search has been selected in this paper for its ease of implementation and fast computation.

#### 2.2.1. Random Forest (RF)

**Figure 1:**
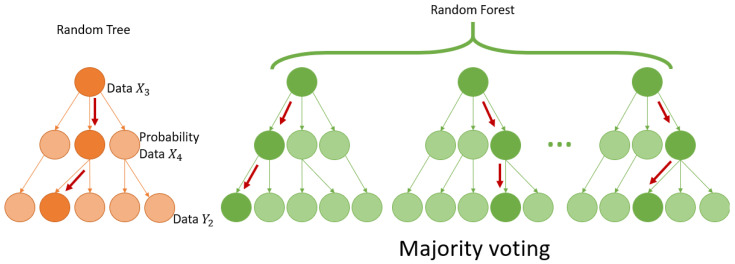
Architecture of a random tree and a Random Forest.

Random Forests extend decision trees described by Breimann et al. in 1984 [34] and expanded upon in 2001 [35]. The dataset is subdivided by decision rules. As shown in Figure 1, it consists of the following:

1. Constructing a multiple decision tree using different smaller datasets that are randomly drawn from the original set;
2. Obtaining predictions from each individual tree;
3. Identifying the most commonly predicted class through a majority voting mechanism.

The Random Forest (RF) algorithm depends on several hyperparameters that must be defined by the user, including the number of observations randomly assigned to each tree, the number of variables randomly selected at each split, the rule used for splitting, the minimum sample size required within a node, and the total number of trees in the ensemble. As noted in [36], the most critical parameter is the number of variables considered for splitting at each node. Typically, increasing the number of trees enhances performance and reduces the risk of overfitting. While the proportion of data allocated to each tree and the minimum sample size for leaf nodes have a smaller impact, they can still be fine-tuned for better results. RF is widely regarded as user-friendly since it primarily depends on two key parameters: the number of variables randomly selected at each node and the total number of trees in the forest.

#### 2.2.2. Artificial Neural Network (ANN)

**Figure 2:**
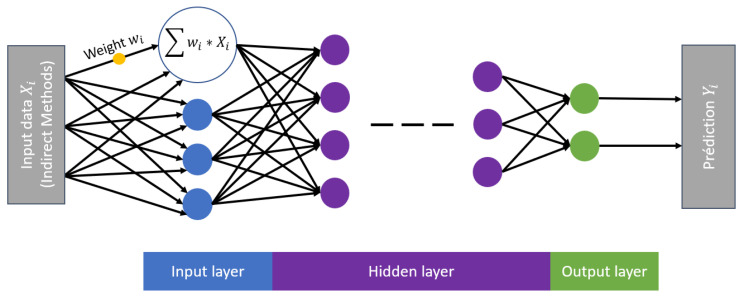
Architecture of an ANN and its basic component.

Artificial Neural Networks can be broadly categorized into three main types: feedforward, feedback, and graph-based networks. This paper specifically focuses on a subtype of feedforward neural networks known as the multi-layer perceptron (MLP). The structure of MLP consists of three distinct layers, as illustrated in Figure 2:

1. Input layer—this layer contains neurons responsible for receiving training data;
2. Hidden layer(s)—one or more intermediate layers composed of neurons that perform mathematical transformations on the input data;
3. Output layer—the final layer that provides the network’s predictions.

In an MLP, data move sequentially from the input layer, through the hidden layers, and finally to the output layer. Each neuron in a given layer is connected to the neurons of the preceding layer through an activation function. This function computes a weighted sum of the input values, adds a bias term, and applies a nonlinear transformation to produce the output. Training an MLP involves optimizing the set of weights and biases to minimize a given loss function. The efficiency of this process depends on several key parameters:

1. The number of hidden layers;
2. The number of neurons per hidden layer;
3. The choice of activation functions (e.g., rectified linear, sigmoid, hyperbolic tangent, etc.)
4. The selection of optimization algorithms (e.g., stochastic gradient descent, particle swarm optimization, evolutionary algorithms, etc.) [37,38,39].

For instance, increasing the number of hidden layers and neurons excessively can lead to overfitting, where the model memorizes training data instead of generalizing well to new inputs. An example of the parameters’ optimization can be found in [40], which compares ANN and the decision tree for the estimation of the concrete strength.

## 3. Results and Discussion

**Figure 3:**
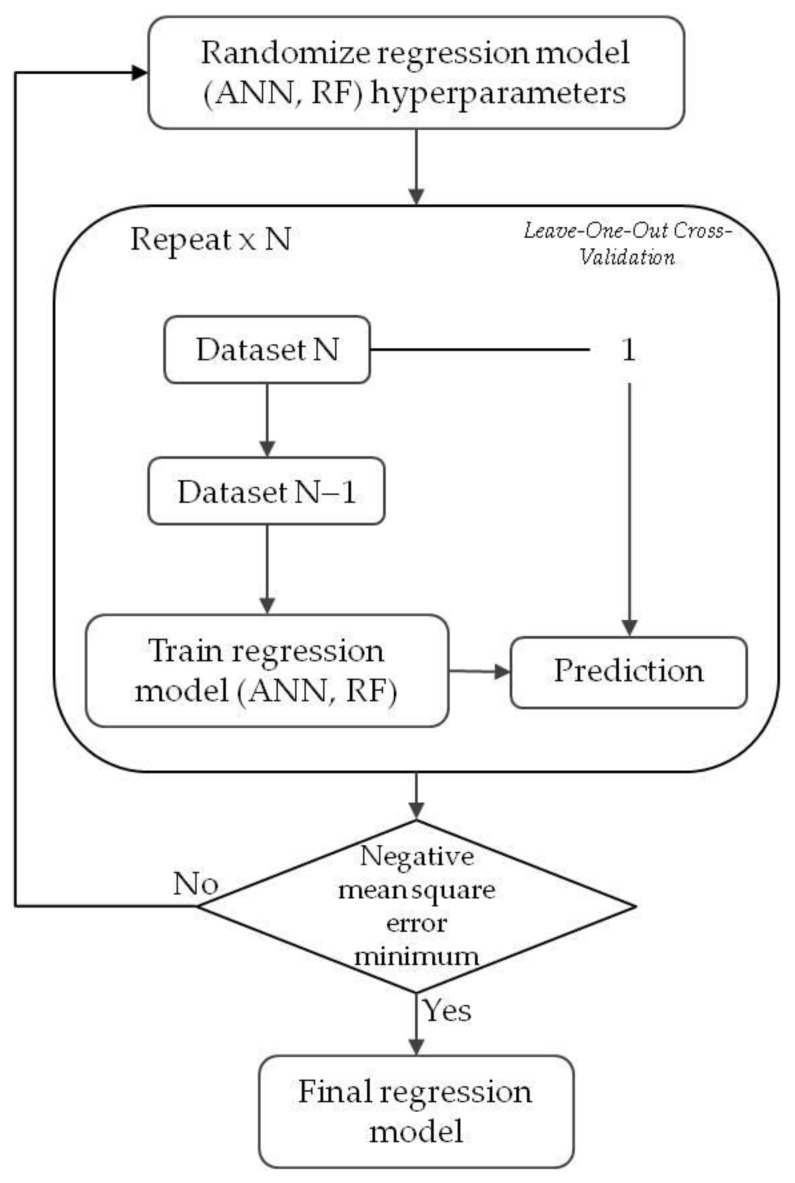
Simplified flowchart to generate a regression model by coupling a randomized search method for hyperparameter tuning with the leave-one-out cross-validation method to estimate the model.

The regression models were developed using the Scikit-learn framework (see Figure 3). The hyperparameters were tuned by using the randomized search method [41], which consists of the following:

1. Randomly sampling a specified number of combinations of hyperparameters from the defined hyperparameter space;
2. Training a model using the training data;
3. Evaluating its performance using cross-validation.

The models’ performance was evaluated using leave-one-out cross-validation [42], a technique where a single data point is removed from the dataset at a time to assess the model’s ability to predict the omitted value. While the dataset consists of 220 samples, which may be considered relatively small, the use of leave-one-out cross-validation ensures that the model is evaluated on each sample independently, thereby maximizing the use of available data and providing reliable performance metrics. The combination of randomized search for hyperparameter optimization and leave-one-out cross-validation provides a robust and well-validated approach. This ensures that the model’s performance is not only optimized but also rigorously evaluated for generalizability.

### 3.1. Validation

There exist many metrics to assess the performance of multi-variable regression models. The coefficient of determination R-squared ($R^{2}$) and Root Mean Squared Error (RMSE) were chosen. The $R^{2}$ score gives the proportion of variation in outputs that is explained by the model. The RMSE score gives the average deviation between the predicted output made by the model and the raw data, assessing the accuracy of the regression model. The normalization process of RMSE consists of dividing the RMSE for each variable by its respective range and provides a measure of prediction accuracy relative to the variability of the data. Table 2 summarizes the results of model performances based on these scores using a leave-one-out cross-validation. The Artificial Neural Network (with the main optimized hyperparameters: three hidden layers and number of neurons by layer (50, 100, 50)) gives the best RMSE and $R^{2}$ scores for the porosity prediction. The RMSE value tells us that the average deviation between the predicted porosity made by the model and the actual porosity is 0.15%. The $R^{2}$ value tells us that the predictor variables in the model are able to explain 99% of the variation in the porosity, suggesting that 99% of the variability in porosity can be attributed to the features included in the model. Random Forest models (with the main optimized hyperparameters trees = 69 and splitting variables = 3) give the best results for water saturation, elastic modulus, and density.

**Figure 4:**
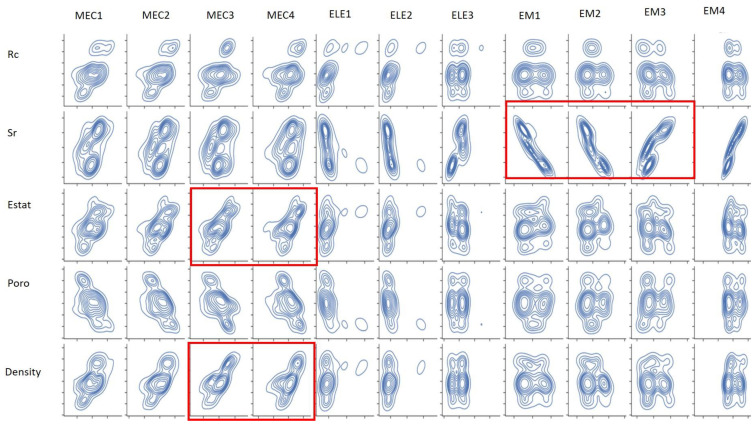
A set of Kernel density estimation pairplots for each pair of mechanical, electromagnetic, and electrical measurements and indicators.

ANNs allow nonlinear relationships to model due to their layered architecture. The pairplots in Figure 4 display smoothed contours representing the probability density of the pairwise variable relationships. As shown in Figure 4, there does not appear to be a linear relationship between the Rc and porosity indicators and the mechanical, electromagnetic, and electrical measurements, which may explain why the ANN outperforms the RF. However, it appears that some mechanical and electromagnetic measurements are linearly related to the Sr, Estat, and density indicators (see the red box in Figure 4). Random Forests often outperform Artificial Neural Networks for linear relationships, which likely explains why RF outperforms ANN for these indicators.

In Table 2, NRMSE and $R^{2}$ seem related since, as $R^{2}$ approaches 1, the NRMSE approaches 0. The NRMSE does not exceed 9% for ANN and 6.5% for RF, which means that the results are of good performance prediction. With an NRMSE of 6.5% for ANN and RF, the density is the parameter that is the most difficult to approach. This can be attributed to the unreliable nature of the density measurement since the assumption that concrete from the same batch has the same density is made. The saturation rate is the parameter that benefits most from the combination of techniques since $R^{2}$ for ANN is weak compared to RF. It brings to attention that, despite their attempt to fit the model to the best, the performance varies between indicators. By selecting the best algorithm, better results can be achieved.

**Figure 5:**
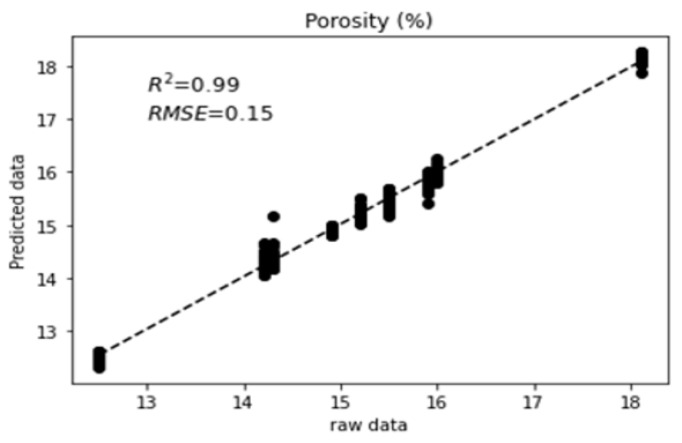
’Leave-one-out’ cross-validation for the Artificial Neural Network model used for the predictions of porosity.

**Figure 6:**
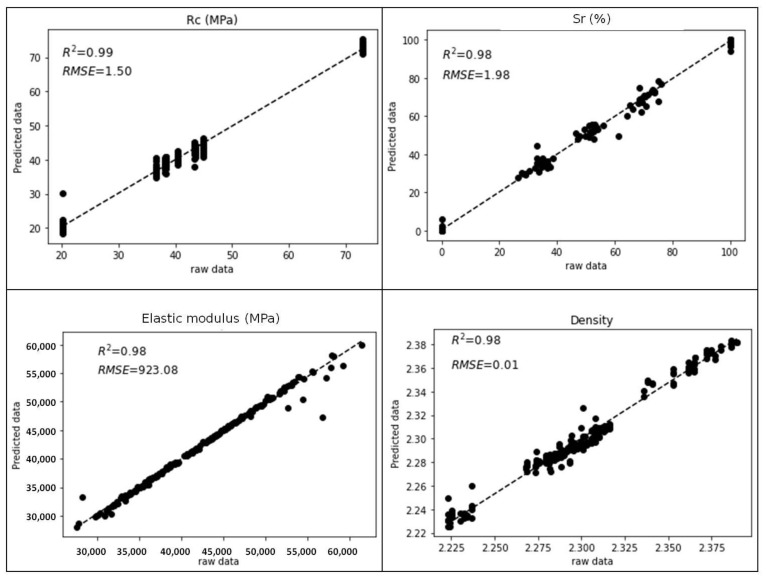
Raw data versus predicted values by using Artificial Neural Network model for Rc and Random Forest models for saturation degree (Sr), elastic modulus and density.

Figure 5 displays and compares the raw data to the ANN model’s predictions of porosity using leave-one-out cross-validation. Each data point is used as a test case once the model is trained on the remaining data. Figure 6 displays the plots of the predicted values of the concrete compressive strength, saturation rate, elastic modulus, and density versus raw data, according to the best models in Table 2.

Previous works present promising results regarding the use of Machine Learning to assess the condition of infrastructures. The performance of ANN or RF models developed through Machine Learning is certainly constrained by the quantity and quality of the data. Indeed, poor quality or quantity of data could lead to underfitting or overfitting. Overfitting can be caused by the model learning noise in the training set when the set is too small or by the addition of too many inputs, which increases the complexity of the prediction. While a large number of inputs can enhance accuracy, the added complexity can lead to fluctuations across different training datasets. The quality of the dataset is also important, as algorithms tend to memorize noise. A balance must be attained between consistency and accuracy, which can be achieved by controlling the model’s complexity and the quality of the dataset. This can be accomplished by reducing the network size or by stopping the algorithm before it starts to learn noise from the data [43].

The results show good precision because of the quantity of observables, but it also leads to increased complexity that could be detrimental to the consistency of those models. They are versatile, as they can be enriched and updated whenever new data become available. Therefore, with each new data collection campaign concerning both the diversity of structures and measurements, the database is enriched, and the models can be updated, enhancing their predictions and broadening their scope of application. To preserve this richness and versatility, feature importance analysis and local explanation could be used to pinpoint the contribution of each observable and select a few.

### 3.2. Feature Importance Analysis and Local Explanation

Feature importance analysis and model-agnostic methods for local explanations are two complementary techniques that enhance the interpretability of Machine Learning models. Feature importance analysis provides insights into the overall behavior of a model by identifying the most influential variables, whereas model-agnostic methods focus on explaining individual predictions, offering reasoning behind the model’s decisions for specific cases. LIME (Local Interpretable Model-agnostic Explanations), PDPs (Partial Dependence Plots), ICE (Individual Conditional Expectations), or SHAP (SHapley Additive exPlanation) are tools and techniques used to interpret and explain Machine Learning models [37]. LIME consists of perturbing the input data and observes changes in predictions to approximate the decision boundary locally. It only focuses on local explanations for individual predictions, which are difficult to generalize for global understanding. PDPs consist in averaging predictions over all instances in the dataset while varying the values of the feature(s) of interest and keeping other features fixed. ICE extends PDPs by showing how a feature affects the prediction for individual instances. They assume independence between features, which may lead to misleading results for correlated features. Based on game theory, SHAP calculates SHapley values to fairly distribute the output of a model across its input features. Unlike methods like PDP, ICE, or LIME, SHAP values are capable of capturing interactions between features. They can be applied in both contexts, independent of the model type, to explain the contribution of each variable to a prediction and to assess alignment with domain expert knowledge [21]. The SHapley Additive exPlanations (SHAP) Python library (version 0.44.1; https://github.com/shap/shap, accessed 10 September 2024) was used to interpret the models.

**Figure 7:**
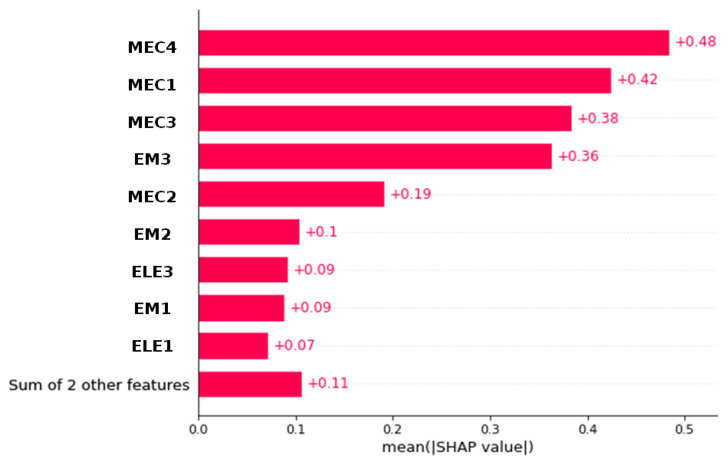
Global importance of variables on the prediction of porosity averaging the absolute value of the SHAP values with the Artificial Neural Network model.

Figure 7 displays the variables ordered by the degree to which they influenced the Artificial Neural Network model’s prediction of porosity, based on the average of the absolute SHAP values of each variable. It shows that variables MEC4, MEC2, MEC3, and EM3 are the most important feature (representing more than 70% of the mean SHAP value total), followed by the MEC1, EM2, ELE3, EM1, etc. Variable MEC4 (i.e., vibratory frequency) has the highest average absolute meaning that variations in the vibration frequency have the greatest influence on predicting porosity.

The measurement methods with results that will most influence the model’s prediction of porosity (which contributes the most) are, thus, acoustic methods with MEC3 (UPV), MEC4 (Impact Echo), and surface wave velocity (MEC2). Electromagnetic (EM) methods with time of propagation measurement are more influenced than peak-to-peak amplitude analysis. Electrical resistivity seems to have less contribution because of its double physical dependency to saturation’s degree and porosity of concrete. The permittivity of concrete is more affected by moisture content variations; that is why the velocity of the EM waves EM2 and EM3 (which are physically related to the permittivity) contributes more than resistivity.

For decision-making, the results of SHAP values distinguish necessary measurement from those not important. Table 3 shows the performances of ANN when fewer observables are used using the SHAP value to select the more significant parameters. For example, the performance remains acceptable with the observables of MEC1, MEC3, MEC4, and EM3. This means that operators can prioritize MEC1, MEC3, MEC4, and EM3 measurements in future inspections. By focusing on these measurements, which are shown to have a stronger influence on the model’s output, engineers and operators may conduct more targeted inspections, reducing time and costs without compromising safety. This prioritization allows NDT analysis to allocate resources more effectively, ensuring that the most impactful tests are performed first. By showing which features drive prediction, SHAP analysis demystifies the model’s decision process, allowing engineers, regulators, and other stakeholders to understand and validate AI-based recommendations. For critical infrastructure applications, this transparency is essential, as it helps ensure that AI-driven insights are reliable, understandable, and actionable, paving the way for wider AI adoption in safety-critical fields.

**Figure 8:**
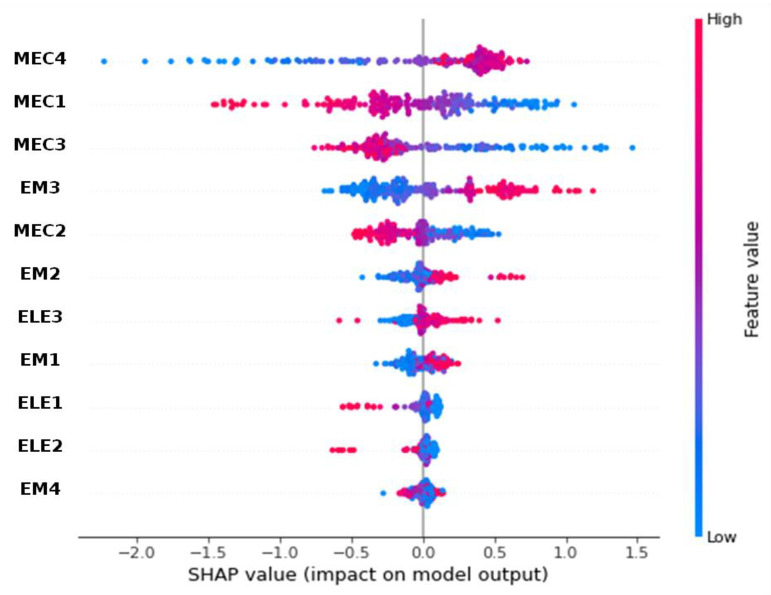
SHAP values showing the impact of various features (MEC4 to EM4) on the model output of porosity (with the Artificial Neural Network model). *y*-axis represents the features ranked by their average absolute SHAP values (see Figure 7). *x*-axis represents SHAP values. The color gradient from blue to pink represents the feature value from low to high, with SHAP values on the *x*-axis indicating the impact magnitude and direction for each feature. Lower measured vibratory frequency (MEC4) leads to a lower predicted porosity. Conversely, higher velocity of surface wave propagation (MEC2) leads to a lower predicted porosity.

Figure 8 displays the SHAP values for all variables for the ANN model’s predictions of porosity. A red point means a higher value of a variable, whereas a blue point represents a lower value. The magnitude of the SHAP value indicates the strength of the variable’s effect on the prediction. A value closer to zero suggests a weaker influence on the prediction, regardless of whether it is positive or negative. A higher distribution of SHAP values (i.e., concentrated dots) suggests that the variable has a more consistent effect on the model’s predictions. For instance, low vibratory frequencies (MEC4) generate negative SHAP values on the porosity, meaning that a lower measured vibratory frequency leads to a lower predicted porosity. Conversely, higher velocity of surface wave propagation (MEC2) leads to a lower predicted porosity. This means if the measured velocity tends to increase or have high values, it will reinforce a prediction of low porosity. If the measurement of MEC2 is high, this will reinforce the fact that porosity is low. However, if the velocity becomes lower, then porosity will be higher. The SHAP values of variables around zero indicate that these variables have a weak influence on the model’s prediction. EM4, ELE2, ELE1, and EM1 do not contribute information to the prediction of porosity.

**Figure 9:**
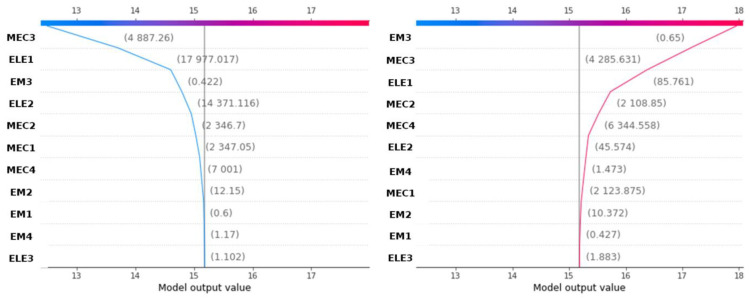
SHAP decision plot: explanation of the contribution of variables for two samples of the dataset leading to the predicted porosity of 12.5% on the left (reps. 17.95% on the top right). On the left, the chart displays how each feature decreases the model output value, while on the right, each feature increases it. The numbers next to each feature represent their specific contributions to the model output value.

Figure 9 displays the decision plots about porosity prediction (12.5% on the left and 17.95% on the right) for two different samples of the dataset. The *x*-axis indicates the model’s output (predicted porosity), while each line shows the cumulative SHAP value representing the total effect of the variables for a specific data point. Beginning from the model’s average prediction (15.18%), with no variable considered, the lines shift left (for negative influence) or right (for positive influence), depending on the variable’s impact on porosity prediction. The endpoint of each line corresponds to the final predicted value. The values in brackets reflect the variable values for each sample. For example, in the left plot of Figure 9, the model identifies that an MEC3 value of 4887.26 m/s, an ELE1 of 17,977.02 Ohm.m, and an EM3 of 0.422 ns all negatively contribute to the porosity prediction. Conversely, Figure 9 (on the right) shows that the model determined an EM3 of 0.65 ns, MEC3 of 4285.63 m/s, and ELE1 of 85.76 Ohm.m, positively influencing the prediction of porosity. By identifying which features have the highest contributions (positive or negative), engineers can prioritize which factors to investigate when anomalies occur. For example, we assumed that a defect occurs for porosity greater than 17%; when a high MEC3 value (see Figure 9) shows a large positive influence on the porosity, it might indicate that high mechanical stress is contributing significantly to potential damage or defect detection, allowing engineers to focus on stress-related factors in their inspections. The decision plot in Figure 9 allows them to verify if the model’s dependencies align with known failure modes or critical parameters in NDT. If unexpected features are dominant, it could signal that the model needs retraining or adjustment. It can be noted that MEC4 has a weak negative influence, which contradicts the previous global results. This result is confirmed by analyzing Figure 8, where some high values can lead to a weak negative influence (seen as red dots close to the left of zero) on the MEC4 line.

### 3.3. Global Importance of Variables on the Prediction

**Figure 10:**
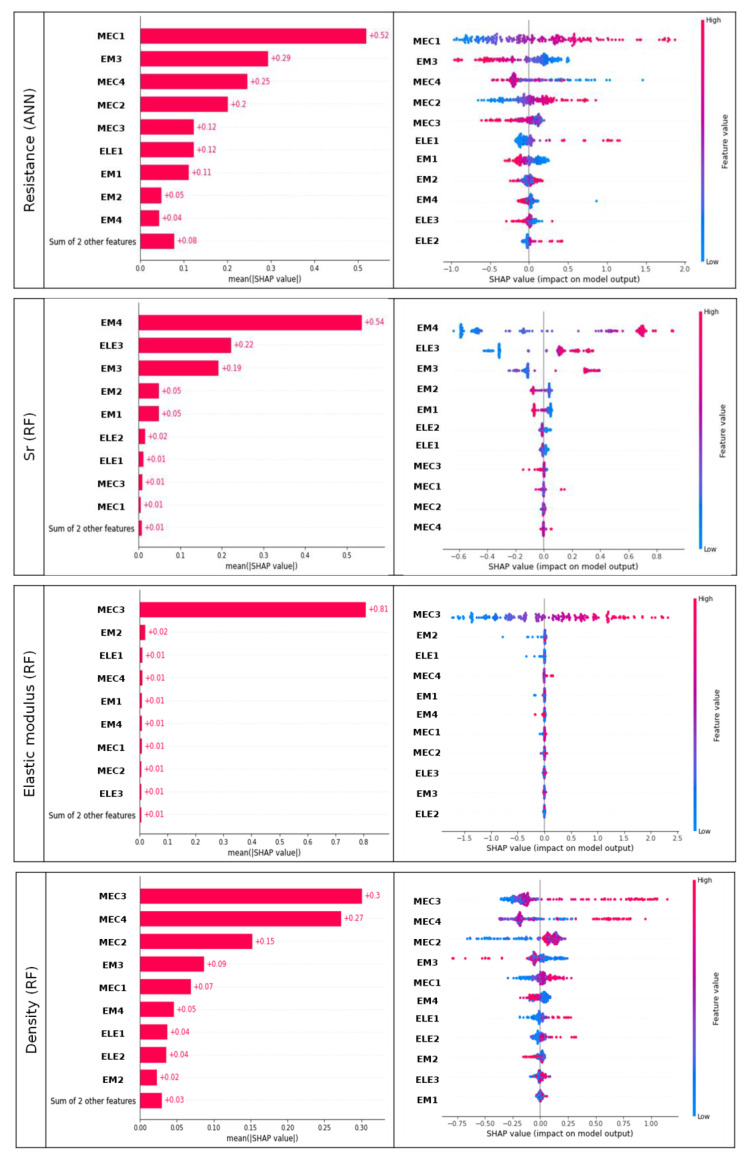
At left: global importance of variables on the prediction of the four different indicators determined by DT (i.e., resistance, degree of saturation, elastic modulus, and density), where *y*-axis represents the features ranked by their average absolute SHAP values. At right: SHAP values on *x*-axis associated with all predictions of the four different indicators, e.g., higher velocity of wave propagation (EM3) leads to a lower predicted resistance by using the ANN model.

Figure 10 displays, on the left (resp. on the right), the variables ordered by the degree to which they influenced (resp. the SHAP values for all variables for) the predictions of the best models for resistance, degree of saturation, elastic modulus, and density.

The measurements of electrical variables do not contribute to the model’s decision-making process regarding the resistance (see Figure 10 on the left). However, the high variability of the SHAP values for MEC2 indicates that the model’s predictions of compressive strength may be less reliable and may be influenced by other factors not captured by that feature. That means that users should exercise caution if they solely rely on this measurement to estimate strength. This suggests that measurements of mechanical and electromagnetic variables alone are sufficient to assess the compressive strength of concrete blocks. Lower mechanical measures and higher electromagnetic measures (see MEC1, MEC2, and EM3 in Figure 10 on the right) lead to lower prediction of resistance. Electromagnetic and electrical measures (see EM3, ELE3, and EM4 in Figure 10 for saturation degree) imply a more consistent impact on the predictions of the degree of saturation, and the low variability of SHAP values suggest that predictions are reliable. The elasticity prediction is only impacted by the velocity of wave propagation (MEC3), indicating that this measure alone seems to be sufficient to provide an assessment of the elastic modulus. However, the variability of the SHAP values for MEC3 compels users to remain cautious about the reliability of the prediction.

In Table 4, we summarize the ranking contribution of measures to the decision support process of the models. The redder the cell is, the more the variable contributes to the model’s decision-making process. The analysis revealed that acoustic measurements, particularly ultrasonic velocity (MEC3 and MEC2) and vibratory frequency (MEC4), were the most influential features across multiple predictions. Electromagnetic measures also played a crucial role, whereas electrical resistivity had a lower impact on model predictions. This suggests that the combination of acoustical and EM NDT methods can be enough for the best prediction of concrete characteristics as compressive strength, porosity, and the degree of saturation.

According to the results, electrical resistivity variables appear to have a lesser contribution to the decision-making process of the models, regardless of the models and predictive variables.

## 4. Conclusions

In this study, we proposed the application of Supervised Machine Learning regression models, specifically the more efficient ones which are Random Forests (RF) and Artificial Neural Networks (ANN), to predict critical properties of concrete infrastructures based on Non-Destructive Testing (NDT) data. The models were trained using a comprehensive dataset comprising various non-destructive acoustical, electrical, and electromagnetic measurements, aiming to provide accurate and explainable predictions of concrete characteristics such as porosity, compressive strength, degree of saturation, elastic modulus, and density. The following conclusions can be drawn:

- The obtained results demonstrate that both RF and ANN models can effectively predict concrete properties from NDT with high accuracy. The study highlights that, while ANN models provided the best performance for porosity predictions, RF models are better in predicting degree of saturation, elastic modulus, and density.
- A significant contribution of this work is the use of SHapley Additive exPlanations (SHAP) to interpret the models’ predictions, and trustworthiness of the Machine Learning models, and giving a reliable tool to optimize the number of sensors needed to make a satisfactory prediction. By integrating SHAP into AI using NDT on infrastructures, users can reduce their reliance on DT for more efficient predictive maintenance. This, in turn, would minimize damage and enable long-term monitoring.
- The results also demonstrate that a limited number of NDTs is actually sufficient for making a valid prediction, particularly regarding elasticity. AI can provide a prediction and its quality with a number of NDT that can be optimized using SHAP.
- The application of Machine Learning models in conjunction with NDT methods holds great promise for the future of civil engineering infrastructure maintenance. By leveraging data-driven insights and ensuring model explainability, stakeholders can make more informed decisions, ultimately contributing to the sustainability and resilience of our built environment.

For routine infrastructure maintenance, these models could be used to estimate more than just one durability indicator, as long as the necessary tests on sensors are conducted, providing an alternative to linear regression methods used, for example, in the EN-13791:2019 standard [4]. This could enable a variety of indirect methods to be deployed for Non-Destructive Evaluation. This expansion would require operator training to oversee Machine Learning, data curation, and noise reduction, as well as specific training on those methods.

Further, other methods such as linear models (Ridge logistic regression and Stochastic Gradient Descent (SGD)), kernel machines (Support Vector Machine (SVM)), local methods (K-Nearest Neighbors (KNN) and Decision Trees), and ensemble methods (Gradient Boosting and XGBoost) could be compared and combined using a voting mechanism to estimate the system’s state. Future work should also focus on further refining these models and expanding their applicability to a broader range of infrastructures and conditions, ensuring their reliability and effectiveness in real-world scenarios.

## Figures and Tables

**Table 2:** Performances of models.

	Variables								
	Porosity (%)			Sr (%)			Elastic Modulus (MPa)		
Models	RMSE	NRMSE	$R^{2}$	RMSE	NRMSE	$R^{2}$	RMSE	NRMSE	$R^{2}$
ANN	**0.15**	**2.5%**	**0.99**	6.28	9%	0.77	1868.6	5.7%	0.93
RF	0.24	4%	0.97	**1.98**	**2.8%**	**0.98**	**923.08**	**2.8%**	**0.98**
	**Variables**								
	**Rc (MPa)**			**Density (** $kg/m^{3}$ **)**					
Models	RMSE	NRMSE	$R^{2}$	RMSE			NRMSE		$R^{2}$
ANN	**1.5**	**2.5%**	**0.99**	0.01			6.5%		0.97
RF	1.78	3%	0.98	**0.01**			**6.5%**		**0.98**

**Table 3:** Performances of ANN with a limited number of observables determined by the SHAP-value.

	Porosity (%) ANN		
Observables	RMSE	NRMSE	$R^{2}$
(MEC1, MEC2, MEC3, MEC4, ELE1, ELE2, ELE3, EM1, EM2, EM3, EM4)	0.15	2.5%	0.99
(MEC1, MEC2, MEC3, MEC4, EM2, EM3)	0.23	3.83%	0.95
(MEC1, MEC2, MEC3, MEC4, EM3)	0.35	5.83%	0.88
(MEC1, MEC3, MEC4, EM3)	0.41	6.83%	0.83
(MEC1, MEC3, MEC4)	0.47	7.83%	0.77

**Table 4:** Ranking of the contribution of measures to the decision support process of the models (the redder the cell is, the more the variable contributes to the model’s decision-making process).

Rank Contribution		Porosity (%) ANN	Rc (MPa) ANN	Sr (%) RF	Elastic Modulus (MPa) ANN	$\mathrm{Density}\;(kg/m^{3})$ RF
Mechanical	MEC1	2	1	9	7	5
	MEC2	5	4	10	8	3
	MEC3	3	5	8	1	1
	MEC4	1	3	11	4	2
Electrical	ELE1	9	6	7	3	7
	ELE2	10	11	6	11	8
	ELE3	7	10	2	9	10
Electromagnetic	EM1	8	7	5	5	11
	EM2	6	8	4	2	9
	EM3	4	2	3	10	4
	EM4	11	9	1	6	6

## Data Availability

The original contributions presented in the study are included in the article, further inquiries can be directed to the corresponding author.
